# Cirrhosis-Associated RAS-Inflammation-Coagulation Axis Anomalies: Parallels to Severe COVID-19

**DOI:** 10.3390/jpm11121264

**Published:** 2021-12-01

**Authors:** Lukas Hartl, Mathias Jachs, Benedikt Simbrunner, David J. M. Bauer, Georg Semmler, Daniela Gompelmann, Thomas Szekeres, Peter Quehenberger, Michael Trauner, Mattias Mandorfer, Bernhard Scheiner, Thomas Reiberger

**Affiliations:** 1 Division of Gastroenterology and Hepatology, Department of Medicine III, Medical University of Vienna, 1090 Vienna, Austria; lukas.a.hartl@meduniwien.ac.at (L.H.); mathias.jachs@meduniwien.ac.at (M.J.); benedikt.simbrunner@meduniwien.ac.at (B.S.); david.bauer@meduniwien.ac.at (D.J.M.B.); georg.semmler@meduniwien.ac.at (G.S.); michael.trauner@meduniwien.ac.at (M.T.); mattias.mandorfer@meduniwien.ac.at (M.M.); bernhard.scheiner@meduniwien.ac.at (B.S.); 2 Vienna Hepatic Hemodynamic Lab, Division of Gastroenterology and Hepatology, Department of Medicine III, Medical University of Vienna, 1090 Vienna, Austria; 3 Christian Doppler Lab for Portal Hypertension and Liver Fibrosis, Medical University of Vienna, 1090 Vienna, Austria; 4 Division of Pulmonology, Department of Medicine II, Medical University of Vienna, 1090 Vienna, Austria; daniela.gompelmann@meduniwien.ac.at; 5 Department of Laboratory Medicine, Medical University of Vienna, 1090 Vienna, Austria; thomas.szekeres@meduniwien.ac.at (T.S.); peter.quehenberger@meduniwien.ac.at (P.Q.)

**Keywords:** portal hypertension, renin-angiotensin-system, fibrinolysis, inflammation, acute respiratory distress syndrome

## Abstract

(1) Background: Cirrhotic patients have an increased risk for severe COVID-19. We investigated the renin-angiotensin-aldosterone system (RAS), parameters of endothelial dysfunction, inflammation, and coagulation/fibrinolysis in cirrhotic patients and in COVID-19 patients. (2) Methods: 127 prospectively characterized cirrhotic patients (CIRR), along with nine patients with mild COVID-19 (mild-COVID), 11 patients with COVID-19 acute respiratory distress syndrome (ARDS; ARDS-COVID), and 10 healthy subjects (HS) were included in the study. Portal hypertension (PH) in cirrhotic patients was characterized by hepatic venous pressure gradient (HVPG). (3) Results: With increased liver disease severity (Child−Pugh stage A vs. B vs. C) and compared to HS, CIRR patients exhibited higher RAS activity (angiotensin-converting enzyme (ACE), renin, aldosterone), endothelial dysfunction (von Willebrand-factor (VWF) antigen), inflammation (C-reactive protein (CRP), interleukin-6 (IL-6)), and a disturbed coagulation/fibrinolysis profile (prothrombin fragment F1,2, D-dimer, plasminogen activity, antiplasmin activity). Increased RAS activity (renin), endothelial dysfunction (vWF), coagulation parameters (D-dimer, prothrombin fragment F1,2) and inflammation (CRP, IL-6) were significantly altered in COVID patients and followed similar trends from mild-COVID to ARDS-COVID. In CIRR patients, ACE activity was linked to IL-6 (ρ = 0.26; *p* = 0.003), independently correlated with VWF antigen (aB: 0.10; *p* = 0.001), and was inversely associated with prothrombin fragment F1,2 (aB: −0.03; *p* = 0.023) and antiplasmin activity (aB: −0.58; *p* = 0.006), after adjusting for liver disease severity. (4) Conclusions: The considerable upregulation of the RAS in Child−Pugh B/C cirrhosis is linked to systemic inflammation, endothelial dysfunction, and abnormal coagulation profile. The cirrhosis-associated abnormalities of ACE, IL-6, VWF antigen, and antiplasmin parallel those observed in severe COVID-19.

## 1. Introduction

Severe acute respiratory distress syndrome-coronavirus-2 (SARS-CoV-2) causes substantial morbidity and mortality worldwide [1]. Coronavirus disease of 2019 (COVID-19) affects lungs, liver, and the neuronal and intestinal system, leading to acute respiratory distress syndrome (ARDS) and multiorgan failure [2,3]. SARS-CoV-2 infection is closely linked to systemic/hepatic inflammation and thrombosis.

Patients with ARDS due to COVID-19 exhibit severe endothelial damage, pulmonary microangiopathy, and thrombosis [4,5], indicating endothelial dysfunction and a dysregulation of the coagulation system. Furthermore, there has been evidence that SARS-CoV-2 infection can also lead to acute splanchnic vein thrombosis [6]. Proinflammatory cytokines, along with activated macrophages and the complement system, are upregulated in SARS-CoV-2 infection, promoting a pronounced inflammatory reaction that may trigger or amplify SARS-CoV-2-associated vascular disease [7]. Moreover, the renin-angiotensin-aldosterone system (RAS) seems to be critically involved in the pathophysiology of COVID-19. The angiotensin-converting enzyme (ACE) 2 receptor was identified as a potential site of entry into the human cell for SARS-CoV-2 [8]. Consecutive RAS dysregulation with relative upregulation of the ACE/angiotensin (Ang) I/Ang II axis and downregulation of the alternative ACE2/Ang (1–7) system is crucial in patients with COVID-19 [9,10].

Similarly, coagulation imbalance, inflammation, and RAS activation also represent well-known hallmarks of advanced chronic liver disease (ACLD) [11,12,13]. Complex changes in the coagulation and fibrinolysis system and other factors lead to an overall prothrombotic state [12], frequently resulting in non-tumoral portal vein thrombosis or venous thromboembolism [14]. Bacterial translocation-induced systemic inflammation in ACLD is associated with the progression of ACLD and is an important promotor of acute on chronic liver failure (ACLF), a syndrome which is characterized by extrahepatic organ failures and high short-term mortality [15].

Patients with ACLD are at particularly high risk for severe courses of COVID-19 [16,17]; however, the underlying mechanisms remain largely unclear. In the present study, we assessed the RAS, endothelial dysfunction, coagulation/fibrinolysis, as well as inflammation in ACLD and COVID-19 to investigate parallels between ACLD patients and patients with severe COVID-19.

## 2. Materials and Methods

ACLD patients undergoing hepatic venous pressure gradient (HVPG) measurement at the Vienna General Hospital between February 2019 and December 2020 with portal hypertension (CIRR) were included in this study. Hemodynamic parameters including mean arterial pressure (MAP) were assessed at the time of HVPG measurement. Blood samples were withdrawn after the patients had rested for at least 30 min in supine position.

Patients with intake of non-selective beta-blockers, antithrombotic or antiplatelet therapy, with active malignancy, portal vein thrombosis, porto-sinusoidal vascular disease or cardiac cirrhosis, as well as patients after liver transplantation were excluded from the study. If multiple HVPG measurements were performed in the same patient, only the baseline measurement (without vasoactive drugs or before etiological therapy) was used for this study. The ACLD patients were stratified by Child−Turcotte−Pugh (CTP) stage A, B, and C and by HVPG (6–9 mmHg, 10–19 mmHg, ≥20 mmHg). Etiology of ACLD, comorbidities (arterial hypertension, diabetes mellitus, coronary heart disease, heart failure), age, and intake of concomitant medication were recorded.

Moreover, inpatients at the General Hospital of Vienna with COVID-19 (mild-COVID), inpatients with ARDS due to COVID-19 (ARDS-COVID), as well as outpatients without ACLD or COVID-19 (‘healthy subjects’, HS) willing to participate in the study were included.

For HVPG measurement, the right jugular vein was accessed via Seldinger technique using a catheter introducer set (8.5F, Arrow International, Reading, PA, USA). A balloon catheter (7F, HVPG catheter, Pejcl Medizintechnik, Vienna, Austria) was subsequently used for liver vein cannulation [18]. HVPG was measured according to standard operating procedure [19] in clinical routine, as supported by Austrian consensus recommendations [20].

A colorimetric assay (ACE kinetic, Bühlmann, Schönenbuch, Switzerland) was used to determine ACE activity. Plasma concentrations of renin (DiaSorin, Liaision XL, Saluggia, Italy) and aldosterone (DiaSorin, Liaision Aldosterone, Saluggia, Italy) were measured by chemiluminescence-immunoassay. VWF antigen was measured by latex agglutination assay (STA LIATEST vWF, Diagnostica Stago, Asnieres, France). Prothrombin fragment F1,2 and plasminogen activator inhibitor (PAI) were assessed by ELISA (Enzygnost F1+2, Siemens, Marburg, Germany; Technozym PAI-1 Actibind, Technoclone, Vienna, Austria), plasminogen activity, and α-2 antiplasmin (antiplasmin) activity by chromogenic assay (STA Stachrom Antiplasmin and STA Stachrom Plasminogen, Diagnostica Stago). Routine laboratory parameters including D-dimer, C-reactive protein (CRP), and interleukin-6 (IL-6) were assessed by standard laboratory methods.

Categorical variables were presented as number (n) of patients and % of these patients with the characteristic of interest, while continuous data was reported as median and interquartile range (IQR). D’Agostino and Pearson and Shapiro–Wilk normality tests were used to test for normal distribution. Mann-Whitney U test was implemented for comparing non-normally distributed continuous variables between two groups, and Kruskal-Wallis test for comparison of non-normally distributed continuous variables in three or more groups. Dunn’s multiple comparisons test was used as post-hoc test. Group comparisons of categorical variables were computed using Pearson’s Chi-squared or Fisher’s exact test.

Factors associated with ACE plasma levels were assessed using linear regression models including either VWF antigen, prothrombin fragment F1,2, or antiplasmin activity. Parameters that showed a trend (*p* < 0.100) were included in the multivariate model. Multicollinearity was investigated via variance inflation factor (VIF). GraphPad Prism 8 (Graphpad Software, La Jolla, CA, USA) and IBM SPSS 22.0 statistic software (IBM, Armonk, NY, USA) were used for statistical analysis. A two-sided *p*-value of <0.05 was considered as being statistically significant.

The study was approved by the ethics committee (EC) of the Medical University of Vienna (study number: 1461/2020 and 1262/2017). It was performed according to the current version of the Helsinki Declaration. All patients were prospectively included in the study and gave their informed consent before the blood withdrawal. The cirrhotic cohort of this study is part of the Vienna Cirrhosis Study registry, registered under www.clinicaltrials.org (accessed on 22 November 2021) (NCT03267615).

## 3. Results

### 3.1. Patient Characteristics

In total, 127 CIRR patients with male predomination (65.4% male) were included in the study. Median age was 56.6 (IQR 15.5) years. Alcoholic (49.6%), viral (15.0%), and cholestatic liver disease (8.7%) were the main etiologies (Table 1). There were 65 (51.2%), 52 (40.9%), and 10 (7.9%) patients in CTP stages A, B, and C, respectively. Median MELD was 11 (IQR 6) points. HVPG increased throughout the CTP stages (A: 14 (IQR 9) mmHg vs. B: 19 (IQR 8) mmHg vs. C: 21 (IQR 5) mmHg; *p* < 0.001).

The mild-COVID cohort consisted of nine patients with COVID-19, who were admitted to the Vienna General Hospital due to the viral infection (22.2% male, median age: 57.1 years), while the ARDS-COVID cohort included 11 patients (54.5% male, median age: 57.4 years) and the HS cohort included 10 subjects (60.0% male, median age: 30.2 years) (Table 2).

### 3.2. Parameters of Endothelial Dysfunction and Coagulation/Fibrinolysis in Cirrhotic Patients

**Figure 1:**
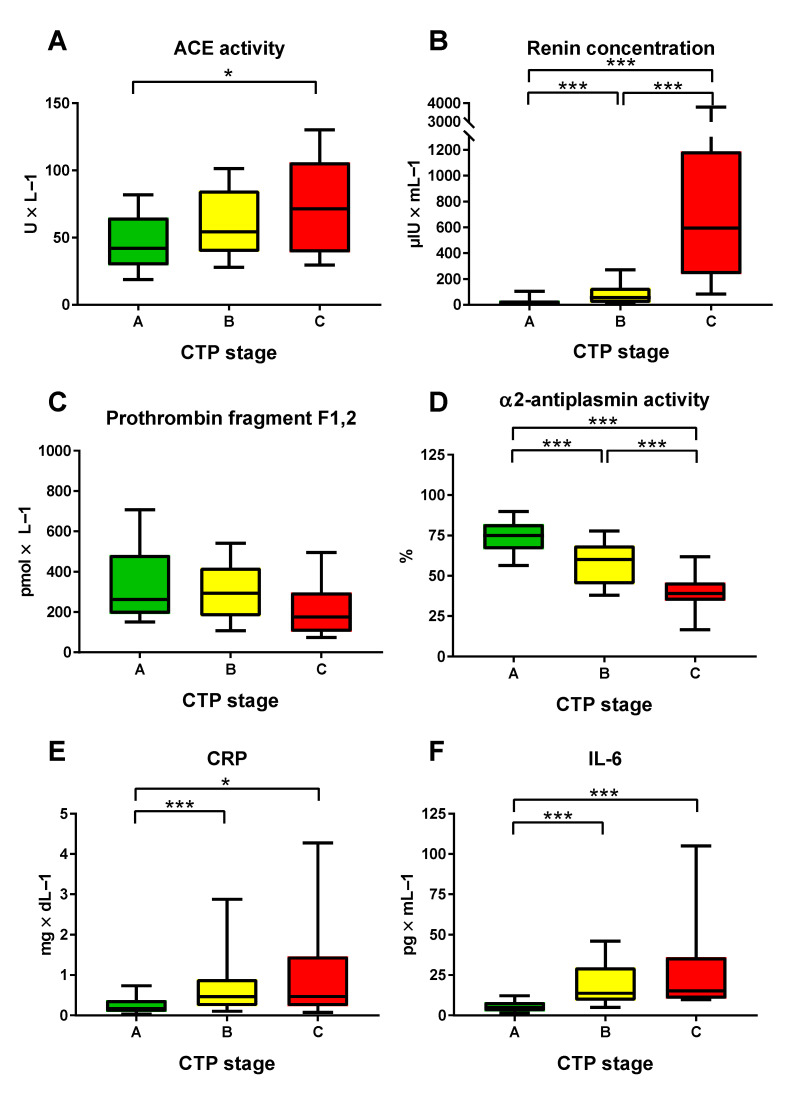
Plasma levels of (**A**) angiotensin-converting enzyme (ACE), (**B**) renin, (**C**) prothrombin fragment F1,2, (**D**) α2-antiplasmin, (**E**) C-reactive protein (CRP), and (**F**) interleukin-6 (IL-6) stratified for CTP score. The borders of the whiskers are the 10th and the 90th percentile. * *p* < 0.050; *** *p* < 0.001.

D-dimer (A: 0.48 (IQR 0.41) µg/mL vs. B: 1.40 (IQR 2.90) µg/mL vs. C: 3.19 (3.08) µg/mL; *p* < 0.001) and VWF antigen (A: 223.4 (IQR 114.0)% vs. B: 307.0 (IQR 133.0)% vs. C: 396.0 (91.0)%; *p* < 0.001) significantly increased with decreasing liver function, while there was a trend for PAI levels (A: 0.89 (1.60) IU/mL vs. B: 0.92 (2.06) IU/mL vs. C: 2.68 (1.73) IU/mL; *p* = 0.149) (Table 1). Antiplasmin activity (A: 74.5 (16.0)% vs. B: 60.0 (24.0)% vs. C: 39.0 (11.0)%; *p* < 0.001), plasminogen activity (A: 75.0 (21.0)% vs. B: 59.0 (12.0)% vs. C: 42.0 (9.0)%; *p* < 0.001), and in tendency prothrombin fragment F1,2 (A: 287.0 (292.0) pmol/L vs. B: 292.5 (241.0) pmol/L vs. C: 176.0 (194.0) pmol/L; *p* = 0.079) decreased with increasing CTP stage (Figure 1). Similar results were obtained when stratifying for the degree of portal hypertension (i.e., HVPG 6–9 mmHg, 10–19 mmHg, ≥20 mmHg) (Figure S1, Table S1).

### 3.3. Parameters of Inflammation and RAS Activation in Cirrhotic Patients

With increasing CTP stage, there was increased inflammation, as indicated by rising CRP (A: 0.18 (0.29) mg/dL vs. B: 0.47 (0.67) mg/dL vs. C: 0.47 (1.23) mg/dL; *p* < 0.001) (Table 1) and IL-6 levels (A: 5.15 (5.65) pg/mL vs. B: 13.70 (20.57) pg/mL vs. C: 15.21 (25.60) pg/mL; *p* < 0.001). Similarly, more pronounced RAS activation was observed in patients with more advanced hepatic dysfunction: ACE (A: 41.3 (35.9) U/L vs. B: 54.4 (45.6) U/L vs. C: 71.3 (66.9) U/L; *p* = 0.006), as well as plasma renin (A: 12.4 (26.7) µIU/mL vs. B: 55.1 (115.9) µIU/mL vs. C: 594.1 (949.8) µIU/mL; *p* < 0.001) and aldosterone concentrations (A: 84.5 (442.0) pg/mL vs. B: 240.0 (442.0) vs. C: 397.0 (238.0) pg/mL; *p* < 0.001).

### 3.4. Differences between COVID-19 Patients with and without ARDS

**Figure 2:**
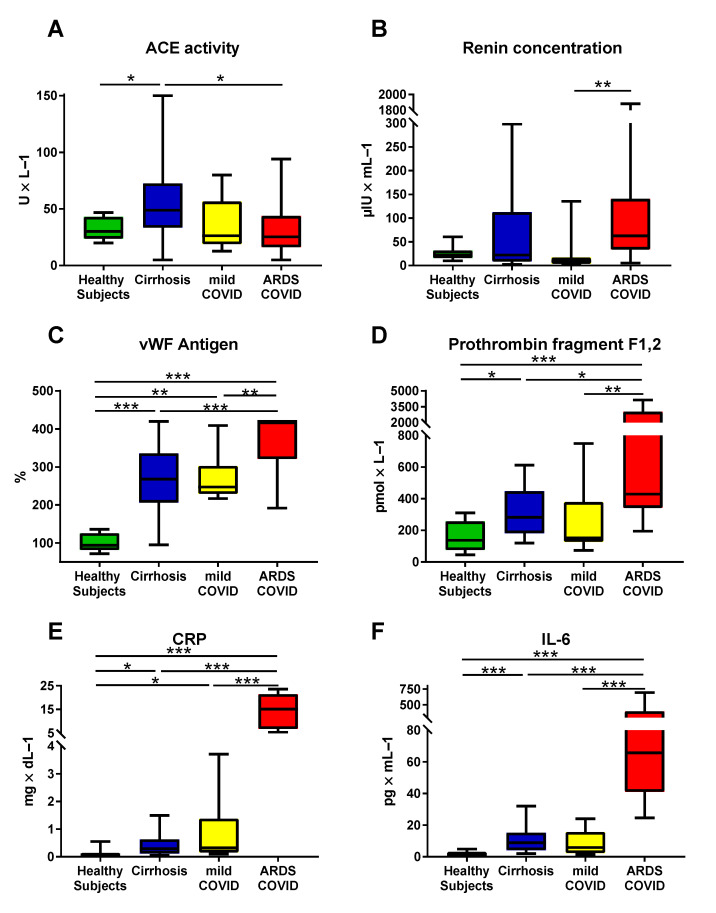
Comparison of plasma levels of (**A**) angiotensin-converting enzyme (ACE), (**B**) renin, (**C**) von Willebrand factor (VWF) antigen, (**D**) prothrombin fragment F1,2, (**E**) C-reactive protein (CRP), and (**F**) interleukin-6 (IL-6) between healthy subjects, cirrhotic patients, patients with mild COVID-19, and patients with ARDS COVID-19. (**A**,**C**) The borders of the whiskers are the minimum and maximum. (**B**,**D**–**F**) The borders of the whiskers are the 10th and the 90th percentile. * *p* < 0.050; ** *p* < 0.010; *** *p* < 0.001.

In ARDS-COVID patients, there were significantly higher levels of plasma renin concentration (mild-COVID: 9.5 (13.1) µIU/mL vs. ARDS-COVID: 62.7 (107.1) µIU/mL; *p* = 0.009), vWF antigen (mild-COVID: 247.5 (73.0) vs. ARDS-COVID: 420.0 (99.0)%; *p* = 0.007), D-dimer (mild-COVID: 0.54 (0.58) µg/mL vs. ARDS-COVID: 2.94 (4.75) µg/mL; *p* < 0.001), prothrombin fragment F1,2 (mild-COVID: 151.5 (252.0) pmol/L vs. ARDS-COVID: 429.0 (2687.0) pmol/L; *p* = 0.006), CRP (mild-COVID: 0.50 (1.42) mg/dL vs. ARDS-COVID: 15.10 (14.69) mg/dL; *p* < 0.001), and IL-6 (mild-COVID: 7.66 (14.74) pg/mL vs. ARDS-COVID: 65.70 (357.90) pg/mL; *p* < 0.001), indicating an increased RAS activation, endothelial dysfunction, coagulation/fibrinolysis activation, and inflammation (Figure 2).

### 3.5. Cirrhotic Patients Resemble Patients with COVID-19 Regarding VWF, Parameters of Coagulation/Fibrinolysis, and Inflammation

Compared to healthy control subjects, cirrhotic patients and COVID-19 patients exhibited markedly increased VWF antigen, while the highest median VWF antigen level occurred in ARDS-COVID patients (Table 2, Figure 2). Both cirrhotic patients and patients with COVID-19 ARDS displayed increased levels of D-dimer and prothrombin fragment F1,2.

Moreover, compared to healthy subjects, cirrhotic and COVID-19 patients had higher levels of CRP and IL-6.

On the other hand, cirrhotic patients exhibited significantly lower antiplasmin activity and plasminogen activity than COVID-19 patients, while ACE activity and plasma aldosterone concentration were higher.

### 3.6. Correlations of ACE and Parameters of Coagulation, Fibrinolysis, and Inflammation in Cirrhosis

**Figure 3:**
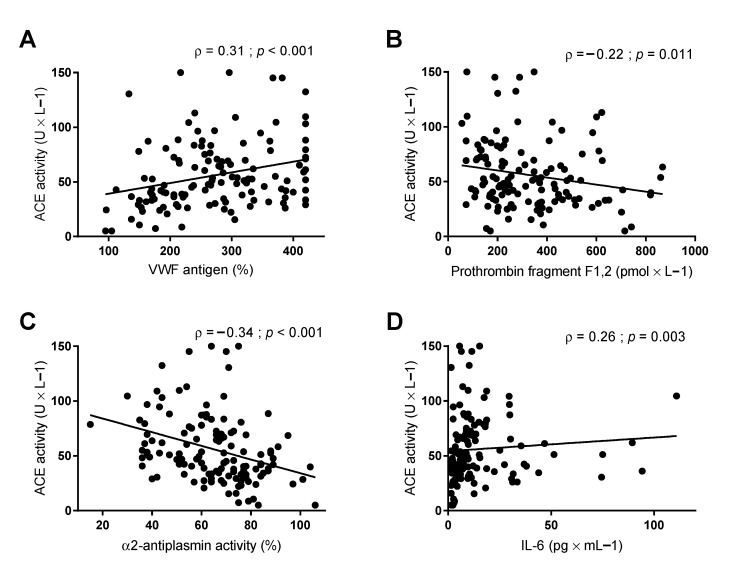
Correlations between plasma levels of angiotensin-converting enzyme (ACE) and plasma levels of (**A**) Von Willebrand factor (VWF) antigen, (**B**) prothrombin fragment F1,2, (**C**) α2-antiplasmin, and (**D**) interleukin-6 (IL-6). Correlations are assessed by Spearman’s Rho.

In CIRR patients, ACE significantly correlated with VWF antigen (ρ = 0.31; *p* < 0.001), D-dimer (ρ = 0.19; *p* = 0.035), prothrombin fragment F1,2 (ρ = −0.22; *p* = 0.011), antiplasmin activity (ρ = −0.34; *p* < 0.001), IL-6 (ρ = 0.26; *p* = 0.003), and HVPG (ρ = 0.26; *p* = 0.003) (Figure 3).

Assessed by multiple linear regression analysis, ACE activity was independently linked to parameters of endothelial dysfunction (i.e., VWF antigen; aB: 0.10; *p* = 0.001), coagulation (i.e., prothrombin fragment F1,2; aB: −0.03; *p* = 0.023), and of fibrinolysis (i.e., antiplasmin activity; aB: −0.58; *p* = 0.006), but not with parameters of inflammation (i.e., CRP and IL-6) (Table 3).

## 4. Discussion

In this study, we thoroughly characterized the state of components of the RAS, coagulation, and inflammation in cirrhotic patients of different disease severity and compared the findings to patients with mild COVID-19 and with COVID-ARDS. Importantly, we identified profound abnormalities of the coagulation system and upregulated systemic inflammation linked to cirrhosis-associated RAS activation that followed similar trends in COVID-19.

By stratifying our ACLD cohort by hepatic dysfunction (i.e., Child stage), as well as for portal pressure (by the diagnostic gold-standard HVPG), we observed marked endothelial dysfunction (i.e., VWF antigen increase) and dysbalanced coagulation state (i.e., D-dimer, prothrombin fragments F1,2 levels) with increasing ACLD severity. At the same time, inflammatory markers (i.e., CRP and IL-6), along with RAS components, were elevated with more severe ACLD, indicating a state of coagulation/fibrinolysis activation linked to RAS activity—that reportedly is upregulated in cirrhosis [21,22].

COVID-19 patients exhibited very similar levels of endothelial dysfunction, coagulation/fibrinolysis, and inflammation parameters, compared to cirrhotic patients, as well as RAS activation, indicated by increased plasma renin concentration. Interestingly, these parameters were also the most important factors differing between mild-COVID and ARDS-COVID. This suggests that cirrhotic patients are in a disadvantageous position when contracting SARS-CoV-2 infection. Importantly, the pronounced RAS activation causing associated coagulation imbalance and a proinflammatory state in Child−Pugh B/C patients may explain why cirrhotic patients have been reported to be particularly susceptible to severe courses of COVID-19 [16,17].

The RAS is intricately involved in cirrhosis development and progression [23]. Importantly, RAS activation leads to worsening of hyperdynamic circulation, making increased plasma renin a predictor for poor clinical outcomes [11,24]. Chronic angiotensin II infusions decrease thrombus generation in mice [25] and ACE inhibitors are associated with decreased thrombin generation [26], indicating the prothrombotic role of RAS components.

In COVID-19, especially the non-classical RAS is subject of scientific interest, as SARS-CoV-2 uses the ACE2 receptor for cell entry [27] and it has been shown that ACE2 is elevated in severe COVID-19 during a later phase of the infection (Day 9–11) [28]. It has been hypothesized that in the initial phase of SARS-CoV-2 infection, a RAS imbalance with increased activation of the classical RAS arm (i.e., renin/ACE/angiotensin II axis) leads to alterations in hemostasis and a hyperinflammatory state, triggering COVID-19 [9]. In our cohort, ACE, as well as aldosterone, were not increased in COVID-19 patients. This is in line with a case series suggesting that ACE is not a suitable biomarker for differentiating between mild and severe COVID-19 [29]. The reason for this might be that the included COVID-19 patients were not in the crucial, initial stage of the disease and in fact, it has been shown that during days 0–3 of SARS-CoV-2 infection, the classical RAS, as represented by angiotensin II, is dramatically upregulated in severe COVID-19 [28]. Fittingly, plasma renin concentration was upregulated in SARS-CoV-2 ARDS patients in our study, compared to non-ARDS COVID-19 patients, underlining the pivotal role of RAS activation for the course of COVID-19. Thus, the state of increased RAS activation in cirrhotic patients, already before an infection with SARS-CoV-2, may be an important factor for their susceptibility for severe COVID-19.

COVID-19 is strongly linked to thromboembolic complications, including microvascular thrombosis, but also macrovascular events such as stroke or pulmonary embolism [5,30]. Endothelial damage is a hallmark of COVID-19, which may be directly caused by the virus, but also by the upregulation of proinflammatory cytokines during the immune response [31]. Accordingly, VWF antigen was found to be increased in lung tissue of SARS-CoV-2−infected macaques [7]. In cirrhosis, VWF antigen has been established as a valuable parameter for risk stratification, indicating endothelial dysfunction and inflammation [32,33,34,35,36,37]. In our study, VWF antigen levels were elevated both in patients with cirrhosis and COVID-19. While patients with cirrhosis and mild-COVID exhibited comparable levels, ARDS-COVID patients had even higher VWF antigen levels, with most values being above the upper limit of quantification of the assay (>420%).

Similarly, D-dimer and prothrombin fragment F1,2 were also increased both in cirrhosis and COVID-19, signifying an activation of coagulation and fibrinolysis, with the highest values in ARDS-COVID patients. Interestingly, prothrombin fragment F1,2 levels were highest in Child-A patients, indicating coagulation dysregulation already in an early stage of cirrhosis. Elevated D-dimer was identified as a marker of poor prognosis in patients with COVID-19 [38]. The high incidence of thromboembolic events in severe COVID-19 was reportedly linked to a dysregulation of thrombin production [30]. In cirrhosis, levels of D-dimer and prothrombin fragment F1,2 levels are altered with disease severity, suggesting increased activation of the coagulation cascade [39,40]. Thus, similar mechanisms likely contribute to coagulation activation in cirrhosis and COVID-19.

Furthermore, high plasmin levels are observed in liver cirrhosis, likely due to decreased antiplasmin and elevated tissue-type plasminogen activator activity [40]. Importantly, ACE negatively correlated with antiplasmin activity in our cirrhosis patients, thus possibly contributing to increased levels of plasmin. In COVID-19, plasmin seems to be of high relevance, as it may prime the SARS-CoV-2 glycoprotein 2, facilitating cell entry of viral particles [41]. Thus, elevated plasmin in liver cirrhosis may lead to more severe courses of COVID-19 due to easier entry of SARS-CoV-2 into the host cells.

Finally, both liver cirrhosis and COVID-19 were associated with increased systemic inflammation. As an infectious disease, COVID-19 triggers a complex immune response, leading to a pronounced (and sometimes excessive) inflammatory reaction [42]. Again, RAS activation and ACE2 downregulation contribute by promotion of proinflammatory mediators [43]. In cirrhosis, with progression of ACLD, there is a stepwise increase in inflammatory mediators. Among decompensated patients, an increase in systemic inflammation is observed from stable disease to ACLF [44] and bacterial infections are regarded as a precipitating event for ACLF [45,46].

Elevation of inflammatory parameters in cirrhosis may be due to gut-derived bacterial translocation [15]; however, the upregulated RAS may once again be crucially involved in the development of this pro-inflammatory state, exacerbating inflammatory signals via upregulation of angiotensin II.

Our study has limitations: The cohorts were not age- and sex-matched. While cirrhotic patients were mainly male, the COVID-19 cohort was mainly female. Moreover, the healthy control group was significantly younger than the other groups, which may have impacted laboratory tests such as VWF, which increases with age [47]. However, the ACLD severity-induced increases are considerably more pronounced than those due to conventional determinants such as age or ABO blood type [48]. Moreover, a small sample size in the mild-COVID, ARDS-COVID, and HS cohorts limits the conclusions that can be drawn from comparisons. In addition, the study was designed as a snapshot, phenotyping the different cohorts at a single time point, not capturing longitudinal changes. Furthermore, the study did not include cirrhotic patients with COVID-19. Consequently, a susceptibility for severe COVID-19 among cirrhotic patients cannot be proven by our data, but is strongly suggested by results from previous studies [16,49]. Thus, prospective longitudinal studies are required to link the laboratory parameters in cirrhotic patients to a risk of severe COVID-19. Finally, our study cannot establish a causative association between the observed laboratory changes and the increased risk of severe courses of COVID-19 in patients with cirrhosis.

## 5. Conclusions

In conclusion, we comprehensively investigated the role of the RAS, endothelial dysfunction, coagulation, and inflammation both in patients with cirrhosis and with COVID-19. Importantly, we found striking similarities between cirrhotic patients and COVID-19 patients, as both groups suffer from endothelial dysfunction, coagulation/fibrinolysis activation, and a systemic pro-inflammatory state. The “baseline-upregulation” of the RAS in the setting of advanced cirrhosis may facilitate SARS-CoV-2 cell entry due to decreased antiplasmin activity and consecutively elevated plasmin levels. The molecular mechanisms driven by cirrhosis-associated RAS activation—including the induction of an imbalanced coagulation profile, endothelial dysfunction, and systemic inflammation—may mechanistically explain a susceptibility to severe COVID-19 in cirrhotic patients.

## Figures and Tables

**Table 1:** Patient characteristics of cirrhotic patients (CIRR) and comparison between CTP stages.

Patient Characteristics	All Patients (*n* = 127)	CTP Stage			*p*-Value
		A (*n* = 65)	B (*n* = 52)	C (*n* = 10)	
Sex, male/female (% male)	83/44 (65.4%)	40/25 (61.5%)	37/15 (71.2%)	6/4 (60.0%)	0.518
Age, years (IQR)	56.6 (15.5)	57.7 (16.4)	54.3 (16.3)	49.9 (7.4)	0.076
Etiology of CLD					**0.002**
ALD, *n* (%)	63 (49.6%)	24 (36.9%)	30 (57.7%)	9 (90.0%)	
Viral, *n* (%)	19 (15.0%)	16 (24.6%)	3 (5.8%)	0 (0.0%)	
MIX (ALD/Viral), *n* (%)	5 (3.9%)	1 (1.5%)	4 (7.7%)	0 (0.0%)	
NASH, *n* (%)	8 (6.3%)	8 (12.3%)	0 (0.0%)	0 (0.0%)	
Cholestatic, *n* (%)	11 (8.7%)	7 (10.8%)	4 (7.7%)	0 (0.0%)	
Other, *n* (%)	21 (16.5%)	9 (13.8%)	11 (21.2%)	1 (10.0%)	
MELD, median (IQR)	11 (6)	9 (2)	15 (5)	19 (6)	**<0.001**
Decompensated ACLD, *n* (%)	80 (63.0%)	25 (38.5%)	45 (86.5%)	10 (100.0%)	**<0.001**
Severe/refractory ascites, *n* (%)	9 (7.1%)	0 (0.0%)	8 (15.4%)	1 (10.0%)	**0.005**
History of bleeding, *n* (%)	16 (12.6%)	7 (10.8%)	8 (15.4%)	1 (10.0%)	0.731
Hepatic venous pressure gradient, median (IQR)	18 (9)	14 (9)	19 (8)	21 (5)	**<0.001**
HVPG 6–9 mmHg, *n* (%)	65 (51.2%)	13 (20.0%)	3 (5.8%)	0 (0.0%)	**0.004**
HVPG 10–19 mmHg, *n* (%)	52 (40.9%)	37 (56.9%)	24 (46.2%)	3 (30.0%)	
HVPG ≥20 mmHg, *n* (%)	10 (7.9%)	15 (23.1%)	25 (48.1%)	7 (70.0%)	
Albumin, g × L^−1^ (IQR)	36.5 (7.6)	39.6 (4.9)	33.8 (6.1)	27.3 (8.4)	**<0.001**
Bilirubin, mg × dL^−1^ (IQR)	1.21 (1.26)	0.87 (0.49)	1.81 (1.60)	3.34 (1.25)	**<0.001**
INR, median (IQR)	1.4 (0.3)	1.3 (0.2)	1.4 (0.2)	1.9 (0.7)	**<0.001**
Creatinine, mg × dL^−1^ (IQR)	0.72 (0.25)	0.74 (0.23)	0.75 (0.37)	0.62 (0.20)	0.384
Sodium, mmol × L^−1^ (IQR)	139.0 (5.0)	140.0 (2.0)	137.0 (4.0)	134.5 (7.0)	**<0.001**
Angiotensin converting enzyme, U × L^−1^ (IQR)	48.9 (39.2)	41.3 (35.9)	54.4 (45.6)	71.3 (66.9)	**0.006**
Plasma renin concentration, µIU × mL^−1^ (IQR)	22.0 (103.9)	12.4 (26.7)	55.1 (115.9)	594.1 (949.8)	**<0.001**
Plasma aldosterone concentration, pg × mL^−1^ (IQR)	109.5 (247.0)	84.5 (442.0)	240.0 (442.0)	397.0 (238.0)	**<0.001**
D-dimer, µg × mL^−1^ (IQR)	0.62 (1.75)	0.48 (0.41)	1.40 (2.90)	3.19 (3.08)	**<0.001**
Von Willebrand factor antigen, % (IQR)	267.0 (136.0)	223.4 (114.0)	307.0 (133.0)	396.0 (91.0)	**<0.001**
Prothrombin fragment F1,2, pmol × L^−1^ (IQR)	282.0 (265.0)	287.0 (292.0)	292.5 (241.0)	176.0 (194.0)	0.079
Plasminogen activator inhibitor, IU × mL^−1^ (IQR)	0.89 (2.01)	0.89 (1.60)	0.92 (2.06)	2.68 (1.73)	0.149
α-2 antiplasmin activity, % (IQR)	67.0 (24.0)	74.5 (16.0)	60.0 (24.0)	39.0 (11.0)	**<0.001**
Plasminogen activity, % (IQR)	65.5 (21.0)	75.0 (21.0)	59.0 (12.0)	42.0 (9.0)	**<0.001**
C-reactive protein, mg × dL^−1^ (IQR)	0.29 (0.51)	0.18 (0.29)	0.47 (0.67)	0.47 (1.23)	**<0.001**
Interleukin-6, pg × mL^−1^ (IQR)	8.86 (11.1)	5.15 (5.65)	13.70 (20.57)	15.21 (25.60)	**<0.001**

Note: Bold font shows statistically significant differences between groups.

**Table 2:** Characteristics of healthy subjects and patients with cirrhosis, mild-COVID-19, and ARDS-COVID-19.

Patient Characteristics	Healthy Subjects (*n* = 10)	Cirrhosis (*n* = 127)	Mild-COVID (*n* = 9)	ARDS-COVID (*n* = 11)	*p*-Value
Sex, male/female (% male)	6/4 (60.0%)	83/44 (65.4%)	2/7 (22.2%)	6/5 (54.5%)	0.075
Age, years (IQR)	30.2 (15.4)	54.6 (15.9)	57.1 (19.6)	57.4 (20.5)	**<0.001**
Angiotensin converting enzyme, U × L^−1^ (IQR)	30.2 (39.2)	48.6 (39.2)	23.7 (16.3)	25.4 (27.7)	**<0.001**
Plasma renin concentration, µIU × mL^−1^ (IQR)	22.2 (15.8)	22.0 (103.9)	11.1 (14.6)	62.7 (107.1)	0.050
Plasma aldosterone concentration, pg × mL^−1^ (IQR)	174.0 (161.0)	109.5 (247.0)	39.5 (78.0)	49.0 (48.0)	**<0.001**
D-dimer, µg × mL^−1^ (IQR)	0.28 (0.15)	0.62 (1.75)	0.54 (0.58)	2.94 (4.75)	**<0.001**
Von Willebrand factor antigen, % (IQR)	94.0 (44.0)	268.0 (130.0)	247.5 (73.0)	420.0 (99.0)	**<0.001**
Prothrombin fragment F1,2, pmol × L^−1^ (IQR)	137.5 (181.0)	282.0 (265.0)	151.5 (252.0)	429.0 (2687.0)	**<0.001**
Plasminogen activator inhibitor, IU × mL^−1^ (IQR)	1.41 (2.64)	0.89 (2.02)	0.58 (4.76)	0.61 (5.02)	0.135
α-2 antiplasmin activity, % (IQR)	104.5 (13.0)	67.0 (24.0)	110.0 (15.0)	108.0 (27.0)	**<0.001**
Plasminogen activity, % (IQR)	96.0 (14.0)	66.0 (21.0)	100.0 (26.0)	92.0 (46.0)	**<0.001**
C-reactive protein, mg × dL^−1^ (IQR)	0.08 (0.08)	0.29 (0.51)	0.50 (1.42)	15.10 (14.69)	**<0.001**
Interleukin-6, pg × mL^−1^ (IQR)	1.58 (0.19)	8.86 (11.09)	7.66 (14.74)	65.70 (357.90)	**<0.001**

Note: Bold font shows statistically significant differences between groups.

**Table 3:** Assessment of independent determinants of plasma levels of ACE activity in cirrhotic patients by multiple linear regression analysis: (i) univariate analysis; and adjusted models (ii) including Von Willebrand factor antigen, (iii) prothrombin fragment F1,2; or (iv) α-2 antiplasmin activity. Bold font shows statistically significant differences between groups.

	(i)		(ii)		(iii)		(iv)	
	B	*p*	aB	*p*	aB	*p*	aB	*p*
Age, per 10 years	−2.49	0.267	-	-	-	-	-	-
Sex (male)	5.28	0.365	-	-	-	-	-	-
MELD, points	1.76	**0.005**	1.02	0.147	0.69	0.396	0.24	0.769
HVPG, mmHg	0.84	0.069	−0.08	0.882	0.02	0.970	−0.14	0.790
Albumin, g × L^−1^	−1.32	**0.008**	−0.21	0.758	−1.32	0.007	0.24	0.737
Sodium, mmol × L^−1^	−1.13	0.140	-	-	-	-	-	-
Mean arterial pressure, mmHg	−0.09	0.605	-	-	-	-	-	-
Von Willebrand factor antigen, %	0.10	**0.001**	0.10	**0.001**	-	-	-	-
Prothrombin fragment F1,2, pmol × L^−1^	−0.03	**0.027**	-	-	−0.03	**0.023**	-	-
α-2 antiplasmin activity, %	−0.62	**<0.001**	-	-	-	-	−0.58	**0.006**
C-reactive protein, mg × dL^−1^	−1.87	0.551	-	-	-	-	-	-
Interleukin-6, pg × mL^−1^	0.12	0.411	-	-	-	-	-	-

## Data Availability

All authors have access to the data underlying this manuscript. Insight into the data can be granted at any time upon reasonable request.

## Supplementary Materials

The following are available online at https://www.mdpi.com/article/10.3390/jpm11121264/s1, Table S1: Characteristics of cirrhotic patients (cohort 1) and comparison between HVPG strata, Figure S1: Plasma levels of (A) angiotensin-converting enzyme (ACE), (B) renin, (C) prothrombin fragment F1,2, (D) α2-antiplasmin, (E) C-reactive protein (CRP) and (F) interleukin-6 (IL-6) stratified for hepatic venous pressure gradient (HVPG). The borders of the whiskers are the 10th and the 90th percentile.

## References

[1] Atzrodt C.L., Maknojia I., McCarthy R.D.P., Oldfield T.M., Po J., Ta K.T.L., Stepp H.E., Clements T.P. (2020). A Guide to COVID-19: A global pandemic caused by the novel coronavirus SARS-CoV-2. FEBS J..

[2] Wiersinga W.J., Rhodes A., Cheng A.C., Peacock S.J., Prescott H.C. (2020). Pathophysiology, Transmission, Diagnosis, and Treatment of Coronavirus Disease 2019 (COVID-19): A Review. JAMA.

[3] Nardo A.D., Schneeweiss-Gleixner M., Bakail M., Dixon E.D., Lax S.F., Trauner M. (2021). Pathophysiological mechanisms of liver injury in COVID-19. Liver Int..

[4] Ackermann M., Verleden S.E., Kuehnel M., Haverich A., Welte T., Laenger F., Vanstapel A., Werlein C., Stark H., Tzankov A. (2020). Pulmonary Vascular Endothelialitis, Thrombosis, and Angiogenesis in Covid-19. N. Engl. J. Med..

[5] Lax S.F., Skok K., Zechner P., Kessler H.H., Kaufmann N., Koelblinger C., Vander K., Bargfrieder U., Trauner M. (2020). Pulmonary Arterial Thrombosis in COVID-19 With Fatal Outcome: Results From a Prospective, Single-Center, Clinicopathologic Case Series. Ann. Intern. Med..

[6] Buso G., Becchetti C., Berzigotti A. (2021). Acute splanchnic vein thrombosis in patients with COVID-19: A systematic review. Dig. Liver Dis..

[7] Aid M., Busman-Sahay K., Vidal S.J., Maliga Z., Bondoc S., Starke C., Terry M., Jacobson C.A., Wrijil L., Ducat S. (2020). Vascular Disease and Thrombosis in SARS-CoV-2-Infected Rhesus Macaques. Cell.

[8] Zemlin A.E., Wiese O.J. (2020). Coronavirus disease 2019 (COVID-19) and the renin-angiotensin system: A closer look at angiotensin-converting enzyme 2 (ACE2). Ann. Clin. Biochem..

[9] Lanza K., Perez L.G., Costa L.B., Cordeiro T.M., Palmeira V.A., Ribeiro V.T., Simões E.S.A.C. (2020). Covid-19: The renin-angiotensin system imbalance hypothesis. Clin. Sci..

[10] Sarzani R., Giulietti F., Di Pentima C., Giordano P., Spannella F. (2020). Disequilibrium between the classic renin-angiotensin system and its opposing arm in SARS-CoV-2-related lung injury. Am. J. Physiol. Lung Cell Mol. Physiol..

[11] Hartl L., Jachs M., Desbalmes C., Schaufler D., Simbrunner B., Paternostro R., Schwabl P., Bauer D.J.M., Semmler G., Scheiner B. (2021). The differential activation of cardiovascular hormones across distinct stages of portal hypertension predicts clinical outcomes. Hepatol. Int..

[12] Zermatten M.G., Fraga M., Moradpour D., Bertaggia Calderara D., Aliotta A., Stirnimann G., De Gottardi A., Alberio L. (2020). Hemostatic Alterations in Patients With Cirrhosis: From Primary Hemostasis to Fibrinolysis. Hepatology.

[13] Simbrunner B., Mandorfer M., Trauner M., Reiberger T. (2019). Gut-liver axis signaling in portal hypertension. World J. Gastroenterol..

[14] Intagliata N.M., Caldwell S.H., Tripodi A. (2019). Diagnosis, Development, and Treatment of Portal Vein Thrombosis in Patients With and Without Cirrhosis. Gastroenterology.

[15] Trebicka J., Reiberger T., Laleman W. (2018). Gut-Liver Axis Links Portal Hypertension to Acute-on-Chronic Liver Failure. Visc. Med..

[16] Iavarone M., D’Ambrosio R., Soria A., Triolo M., Pugliese N., Del Poggio P., Perricone G., Massironi S., Spinetti A., Buscarini E. (2020). High rates of 30-day mortality in patients with cirrhosis and COVID-19. J. Hepatol..

[17] Boettler T., Newsome P.N., Mondelli M.U., Maticic M., Cordero E., Cornberg M., Berg T. (2020). Care of patients with liver disease during the COVID-19 pandemic: EASL-ESCMID position paper. JHEP Rep..

[18] Ferlitsch A., Bota S., Paternostro R., Reiberger T., Mandorfer M., Heinisch B., Salzl P., Schwarzer R., Sieghart W., Peck-Radosavljevic M. (2015). Evaluation of a new balloon occlusion catheter specifically designed for measurement of hepatic venous pressure gradient. Liver Int..

[19] Reiberger T., Schwabl P., Trauner M., Peck-Radosavljevic M., Mandorfer M. (2019). Measurement of the Hepatic Venous Pressure Gradient and Transjugular Liver Biopsy. J. Vis. Exp. JoVE.

[20] Reiberger T., Puspok A., Schoder M., Baumann-Durchschein F., Bucsics T., Datz C., Dolak W., Ferlitsch A., Finkenstedt A., Graziadei I. (2017). Austrian consensus guidelines on the management and treatment of portal hypertension (Billroth III). Wien. Klin. Wochenschr..

[21] Wilkinson S.P., Williams R. (1980). Renin-angiotensin-aldosterone system in cirrhosis. Gut.

[22] Granzow M., Schierwagen R., Klein S., Kowallick B., Huss S., Linhart M., Mazar I.G., Görtzen J., Vogt A., Schildberg F.A. (2014). Angiotensin-II type 1 receptor-mediated Janus kinase 2 activation induces liver fibrosis. Hepatology.

[23] Sansoè G., Aragno M., Wong F. (2020). Pathways of hepatic and renal damage through non-classical activation of the renin-angiotensin system in chronic liver disease. Liver Int..

[24] Paternostro R., Reiberger T., Mandorfer M., Schwarzer R., Schwabl P., Bota S., Ferlitsch M., Trauner M., Peck-Radosavljevic M., Ferlitsch A. (2017). Plasma renin concentration represents an independent risk factor for mortality and is associated with liver dysfunction in patients with cirrhosis. J. Gastroenterol. Hepatol..

[25] Senchenkova E.Y., Russell J., Almeida-Paula L.D., Harding J.W., Granger D.N. (2010). Angiotensin II-mediated microvascular thrombosis. Hypertension.

[26] Ekholm M., Wallén N.H., Johnsson H., Eliasson K., Kahan T. (2002). Long-term angiotensin-converting enzyme inhibition with ramipril reduces thrombin generation in human hypertension. Clin. Sci..

[27] Wrapp D., Wang N., Corbett K.S., Goldsmith J.A., Hsieh C.L., Abiona O., Graham B.S., McLellan J.S. (2020). Cryo-EM structure of the 2019-nCoV spike in the prefusion conformation. Science.

[28] Reindl-Schwaighofer R., Hödlmoser S., Eskandary F., Poglitsch M., Bonderman D., Strassl R., Aberle J.H., Oberbauer R., Zoufaly A., Hecking M. (2021). ACE2 Elevation in Severe COVID-19. Am. J. Respir. Crit. Care Med..

[29] Kolberg E.S., Wickstrøm K., Tonby K., Dyrhol-Riise A.M., Holten A.R., Amundsen E.K. (2021). Serum ACE as a prognostic biomarker in COVID-19: A case series. APMIS.

[30] McFadyen J.D., Stevens H., Peter K. (2020). The Emerging Threat of (Micro)Thrombosis in COVID-19 and Its Therapeutic Implications. Circ. Res..

[31] Varga Z., Flammer A.J., Steiger P., Haberecker M., Andermatt R., Zinkernagel A.S., Mehra M.R., Schuepbach R.A., Ruschitzka F., Moch H. (2020). Endothelial cell infection and endotheliitis in COVID-19. Lancet.

[32] Mandorfer M., Schwabl P., Paternostro R., Pomej K., Bauer D., Thaler J., Ay C., Quehenberger P., Fritzer-Szekeres M., Peck-Radosavljevic M. (2018). Von Willebrand factor indicates bacterial translocation, inflammation, and procoagulant imbalance and predicts complications independently of portal hypertension severity. Aliment. Pharmacol. Ther..

[33] Ferlitsch M., Reiberger T., Hoke M., Salzl P., Schwengerer B., Ulbrich G., Payer B.A., Trauner M., Peck-Radosavljevic M., Ferlitsch A. (2012). von Willebrand factor as new noninvasive predictor of portal hypertension, decompensation and mortality in patients with liver cirrhosis. Hepatology.

[34] Jachs M., Hartl L., Simbrunner B., Bauer D., Paternostro R., Scheiner B., Schwabl P., Stättermayer A.F., Pinter M., Eigenbauer E. (2021). Decreasing von Willebrand Factor Levels Upon Nonselective Beta Blocker Therapy Indicate a Decreased Risk of Further Decompensation, Acute-on-chronic Liver Failure, and Death. Clin. Gastroenterol. Hepatol..

[35] Starlinger P., Ahn J.C., Mullan A., Gyoeri G.P., Pereyra D., Alva-Ruiz R., Hackl H., Reiberger T., Trauner M., Santol J. (2021). The Addition of C-Reactive Protein and von Willebrand Factor to Model for End-Stage Liver Disease-Sodium Improves Prediction of Waitlist Mortality. Hepatology.

[36] La Mura V., Reverter J.C., Flores-Arroyo A., Raffa S., Reverter E., Seijo S., Abraldes J.G., Bosch J., García-Pagán J.C. (2011). Von Willebrand factor levels predict clinical outcome in patients with cirrhosis and portal hypertension. Gut.

[37] Violi F., Ferro D., Basili S., Saliola M., Quintarelli C., Alessandri C., Cordova C. (1995). Association between low-grade disseminated intravascular coagulation and endotoxemia in patients with liver cirrhosis. Gastroenterology.

[38] Zhou F., Yu T., Du R., Fan G., Liu Y., Liu Z., Xiang J., Wang Y., Song B., Gu X. (2020). Clinical course and risk factors for mortality of adult inpatients with COVID-19 in Wuhan, China: A retrospective cohort study. Lancet.

[39] Zocco M.A., Di Stasio E., De Cristofaro R., Novi M., Ainora M.E., Ponziani F., Riccardi L., Lancellotti S., Santoliquido A., Flore R. (2009). Thrombotic risk factors in patients with liver cirrhosis: Correlation with MELD scoring system and portal vein thrombosis development. J. Hepatol..

[40] Leebeek F.W., Kluft C., Knot E.A., de Maat M.P., Wilson J.H. (1991). A shift in balance between profibrinolytic and antifibrinolytic factors causes enhanced fibrinolysis in cirrhosis. Gastroenterology.

[41] Sansoè G., Aragno M., Wong F. (2021). COVID-19 and Liver Cirrhosis: Focus on the Nonclassical Renin-Angiotensin System and Implications for Therapy. Hepatology.

[42] Paces J., Strizova Z., Smrz D., Cerny J. (2020). COVID-19 and the immune system. Physiol. Res..

[43] Verdecchia P., Cavallini C., Spanevello A., Angeli F. (2020). The pivotal link between ACE2 deficiency and SARS-CoV-2 infection. Eur. J. Intern. Med..

[44] Balcar L., Semmler G., Pomej K., Simbrunner B., Bauer D., Hartl L., Jachs M., Paternostro R., Bucsics T., Pinter M. (2021). Patterns of acute decompensation in hospitalized patients with cirrhosis and course of acute-on-chronic liver failure. United Eur. Gastroenterol. J..

[45] Moreau R., Jalan R., Gines P., Pavesi M., Angeli P., Cordoba J., Durand F., Gustot T., Saliba F., Domenicali M. (2013). Acute-on-chronic liver failure is a distinct syndrome that develops in patients with acute decompensation of cirrhosis. Gastroenterology.

[46] Trebicka J., Fernandez J., Papp M., Caraceni P., Laleman W., Gambino C., Giovo I., Uschner F.E., Jansen C., Jimenez C. (2021). PREDICT identifies precipitating events associated with the clinical course of acutely decompensated cirrhosis. J. Hepatol..

[47] Seaman C.D., Ragni M.V. (2020). The Effect of Age on von Willebrand Factor and Bleeding Symptoms in von Willebrand Disease. Thromb. Haemost..

[48] Scheiner B., Northup P.G., Gruber A.B., Semmler G., Leitner G., Quehenberger P., Thaler J., Ay C., Trauner M., Reiberger T. (2020). The impact of ABO blood type on the prevalence of portal vein thrombosis in patients with advanced chronic liver disease. Liver Int..

[49] Boettler T., Marjot T., Newsome P.N., Mondelli M.U., Maticic M., Cordero E., Jalan R., Moreau R., Cornberg M., Berg T. (2020). Impact of COVID-19 on the care of patients with liver disease: EASL-ESCMID position paper after 6 months of the pandemic. JHEP Rep..

