# Supplementary material for: Cirrhosis-Associated RAS-Inflammation-Coagulation Axis Anomalies: Parallels to Severe COVID-19
Source: J Pers Med. 2021 Dec 1;11(12):1264. doi: 10.3390/jpm11121264 (PMC8709210; doi:10.3390/jpm11121264)

Supplementary Material

Table-S1. Characteristics of cirrhotic patients (cohort 1) and comparison between HVPG strata

Patient characteristics	All patients	HVPG			p-value
	(*n*=127)	6-9mmHg (*n*=16)	10-19mmHg (*n*=64)	≥20mmHg (*n*=47)	
**Sex, male/female (% male)**	83/44 (65.4%)	10/6 (62.5%)	42/22 (65.6%)	31/16 (66.0%)	0.967
**Age, years (IQR)**	56.6 (15.5)	52.8 (17.8)	53.4 (15.4)	57.1 (15.0)	0.285
**Etiology of CLD**					**0.003**
**ALD, n (%)**	63 (49.6%)	3 (18.8%)	27 (42.2%)	33 (70.2%)	
**Viral, n (%)**	19 (15.0%)	5 (31.3%)	10 (15.6%)	4 (8.5%)	
**MIX (ALD/Viral), n (%)**	5 (3.9%)	0 (0.0%)	4 (6.3%)	1 (2.1%)	
**NASH, n (%)**	8 (6.3%)	2 (12.5%)	6 (9.4%)	0 (0.0%)	
**Cholestatic, n (%)**	11 (8.7%)	0 (0.0%)	6 (9.4%)	5 (10.6%)	
**Other, n (%)**	21 (16.5%)	6 (37.5%)	11 (17.2%)	4 (8.5%)	
**MELD, median (IQR)**	11 (6)	8 (2)	10 (5)	14 (7)	**<0.001**
**CTP Score, median (IQR)**	6 (3)	5 (0)	6 (2)	7 (3)	**<0.001**
**Child-A, n (%)**	65 (51.2%)	13 (81.3%)	37 (57.8%)	15 (31.9%)	**0.004**
**Child-B, n (%)**	52 (40.9%)	3 (18.7%)	24 (37.5%)	25 (53.2%)	
**Child-C, n (%)**	10 (7.9%)	0 (0.0%)	3 (4.7%)	7 (14.9%)	
**Albumin, g x L^{-1} (IQR)**	36.5 (7.6)	42.9 (5.3)	37.0 (6.9)	35.7 (7.2)	**<0.001**
**Bilirubin, mg x dL^{-1} (IQR)**	1.21 (1.26)	0.83 (0.68)	1.07 (1.01)	1.62 (1.73)	**0.005**
**INR, median (IQR)**	1.4 (0.3)	1.1 (0.2)	1.3 (0.3)	1.5 (0.3)	**<0.001**
**Creatinine, mg x dL^{-1} (IQR)**	0.72 (0.25)	0.85 (0.27)	0.71 (0.23)	0.67 (0.27)	0.267
**Sodium, mmol x L^{-1} (IQR)**	139.0 (5.0)	140.0 (5.0)	139.0 (4.0)	137.0 (5.0)	**0.004**
**D-dimer, µg x mL^{-1} (IQR)**	0.62 (1.75)	0.37 (0.32)	0.56 (1.48)	1.23 (2.73)	**<0.001**
**Von Willebrand factor antigen, % (IQR)**	267.0 (136.0)	179.5 (58.0)	257.0 (142.0)	319 (131.0)	**<0.001**
**Prothrombin fragment F1,2, pmol x L^{-1} (IQR)**	282.0 (265.0)	384.5 (387.2)	300.0 (269.0)	244.0 (213.0)	0.150
**Plasminogen activator inhibitor, IU x mL^{-1} (IQR)**	0.89 (2.01)	0.89 (1.91)	0.89 (1.78)	0.89 (2.16)	0.535
**α-2 antiplasmin activity, % (IQR)**	67.0 (24.0)	80.0 (16.8)	69.0 (18.5)	57.0 (21.0)	**<0.001**
**Plasminogen activity, % (IQR)**	65.5 (21.0)	83.5 (21.0)	66.0 (21.0)	64.0 (13.0)	**<0.001**
**C-reactive protein, mg x dL^{-1} (IQR)**	0.29 (0.51)	0.09 (0.25)	0.13 (0.44)	0.43 (0.67)	**0.006**

**Interleukin-6, pg x mL^{-1} (IQR)**	8.86 (11.1)	2.85 (2.97)	8.46 (13.51)	9.77 (10.94)	**<0.001**
**Angiotensin converting enzyme, U x L^{-1} (IQR)**	48.9 (39.2)	37.25 (17.3)	43.55 (44.68)	60.40 (29.9)	**0.003**
**Plasma renin concentration, µIU x mL^{-1} (IQR)**	22.0 (103.9)	14.5 (24.6)	18.4 (71.6)	62.1 (206.1)	**0.015**
**Plasma aldosterone concentration, pg x mL^{-1} (IQR)**	109.5 (247.0)	101.5 (99.0)	95.0 (184.0)	204.0 (449.0)	0.158

Figure-S1. Plasma levels of (A) angiotensin-converting enzyme (ACE), (B) renin, (C) prothrombin fragment F1,2, (D) α2-antiplasmin, (E) C-reactive protein (CRP) and (F) interleukin-6 (IL-6) stratified for hepatic venous pressure gradient (HVPG). The borders of the whiskers are the 10th and the 90th percentile.

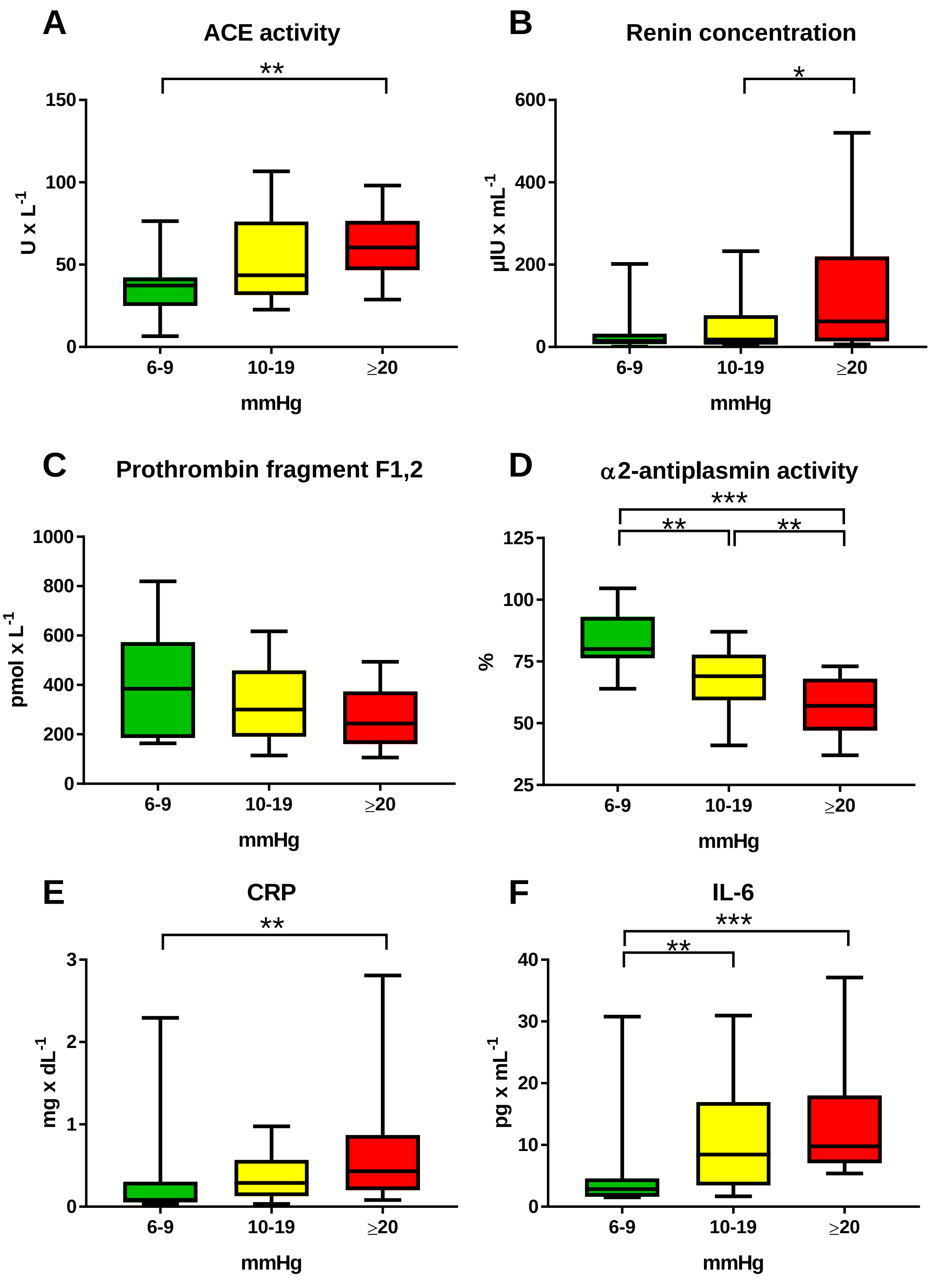

Supplement: Supplementary file 1 [file jpm-11-01264-s001.zip › jpm-1469740-supplementary.pdf]
